# Supplementary material for: Piezo1 regulates the mechanotransduction of soft matrix viscoelasticity
Source: Nat Commun. 2025 Oct 15;16:9155. doi: 10.1038/s41467-025-64185-5 (PMC12528374; doi:10.1038/s41467-025-64185-5)
Supplement: Supplementary file 1 — Supplementary Information [file 41467_2025_64185_MOESM1_ESM.pdf]

Supplementary Materials for

Piezo1 regulates the mechanotransduction of soft matrix viscoelasticity

Mariana A. G. Oliva[1,2], Giuseppe Ciccone[1,2], Gotthold Fläschner[2], Jiajun Luo[1], Jonah L.Voigt[1,3,4], Patrizia Romani[5], Paul Genever[6], Oana Dobre[1], Sirio Dupont[5], Massimo Vassalli[1], Pere Roca-Cusachs[2,7*], Manuel Salmeron-Sanchez[1,2,8*]

Affiliations

[1]Centre for the Cellular Microenvironment (CeMi), University of Glasgow, The Advanced Research Centre, Glasgow, UK

[2]Institute for Bioengineering of Catalonia (IBEC), The Barcelona Institute for Science and Technology (BIST), Barcelona, Spain

[3]Max Planck Institute for Medical Research, Heidelberg, Germany.

[4]Cellular Biomechanics, Faculty of Engineering, Bayreuth University, Bayreuth, Germany.

[5]Department of Molecular Medicine (DMM), University of Padua, Padua, Italy

[6]Department of Biology, University of York, York, UK.

[7]University of Barcelona, Spain

[8]Institució Catalana de Recerca i Estudis Avançats (ICREA), Barcelona, Spain.

Corresponding authors: proca@ibecbarcelona.eu , msalmeron@ibecbarcelona.eu

This PDF file includes:

Supplementary Figs. S1 to S11
Supplementary Note 1
Tables S1 to S4
Supplementary references

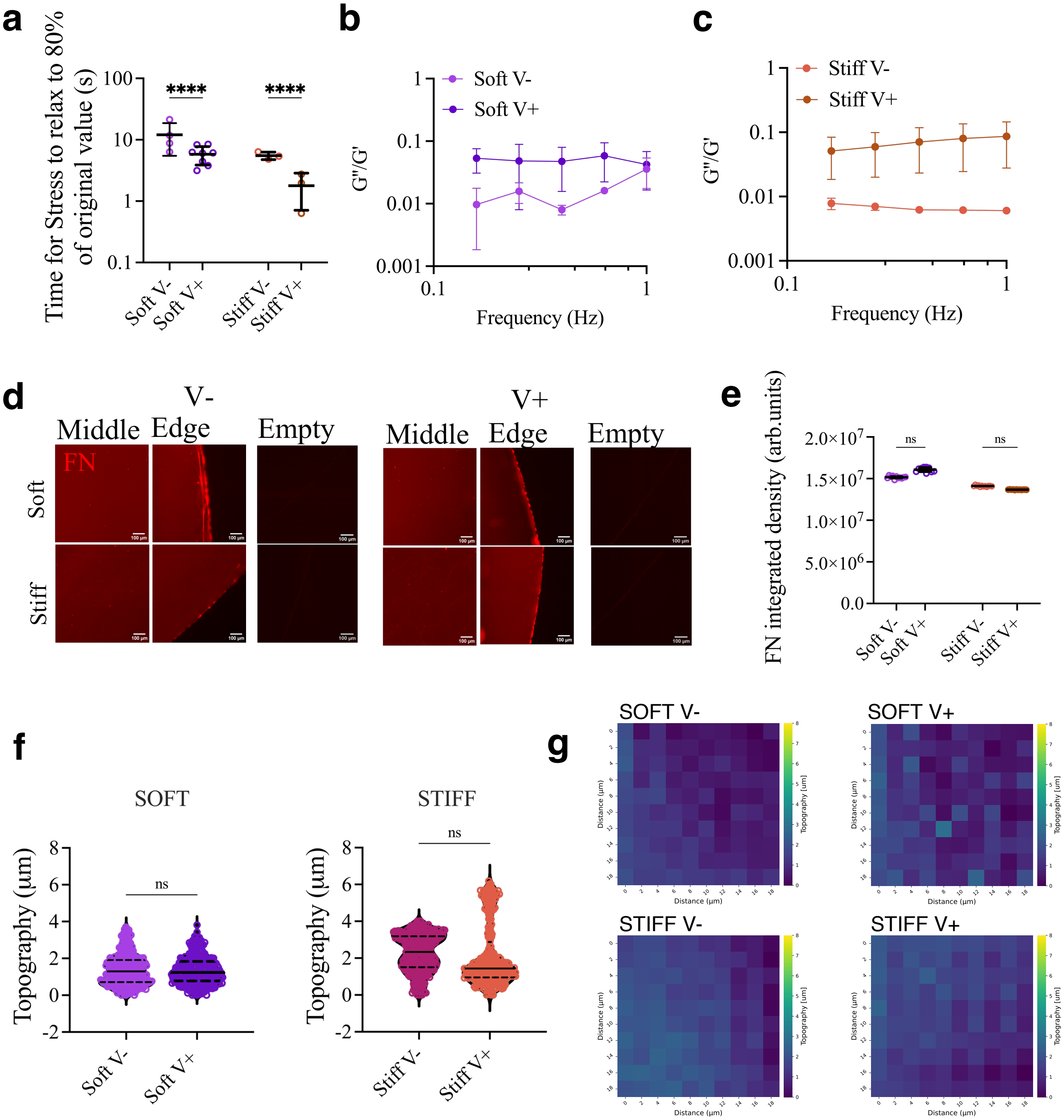

Supplementary Data Fig. 1.

(**a**) Time for stress relaxation to occur to 80% of original stress value (s) plotted for Soft (grey) and Stiff (orange) hydrogel groups. (from left to right n = 61, 87, 62 and 71 curves, each dot represents a map of ≥ 4 single nanoindentation curves each, from N = 3 gels). P values: **** < 0.0001. (**b**) G''/G' plot of bulk rheology frequency sweep measurements performed at 0.1% strain for the soft group. (**c**) G''/G' plot of bulk rheology frequency sweep measurements performed at

0.1% strain for the stiff group. Shown as mean ± SD of N = 3 hydrogels. (**d**) Representative immunofluorescence images of the 10 µg/ml fibronectin coating of the hydrogels, showing the middle and edge of the hydrogel on the glass coverslip. Images of stained non-functionalised hydrogels are also shown in the empty column, for each hydrogel type. Scale bar 100 µm. (**e**) Quantification of the resulting fibronectin staining intensity as individual points, mean ± SD of different areas (n = 3) of each hydrogel (N = 3). From left to right p values: ns = 0.2670 and ns = 0.1923 (**f**) Topography measurements (µm) for soft (left) and stiff (right) hydrogel groups, shown as individual values. In the soft condition, from left to right n = 300 and 285; in the stiff condition, n = 300 and 299 individual measurements shown as a box and violin plot with median and quartiles of N = 3 hydrogels. P values from left to right; ns = 0.4623 and ns = 0.1510 (**g**) Representative 20x20 µm topography maps per hydrogel condition, Statistical analyses were performed using a two-way ANOVA (**a**), Kruskal-Walli's test (**e**) and unpaired t-tests (**f**).

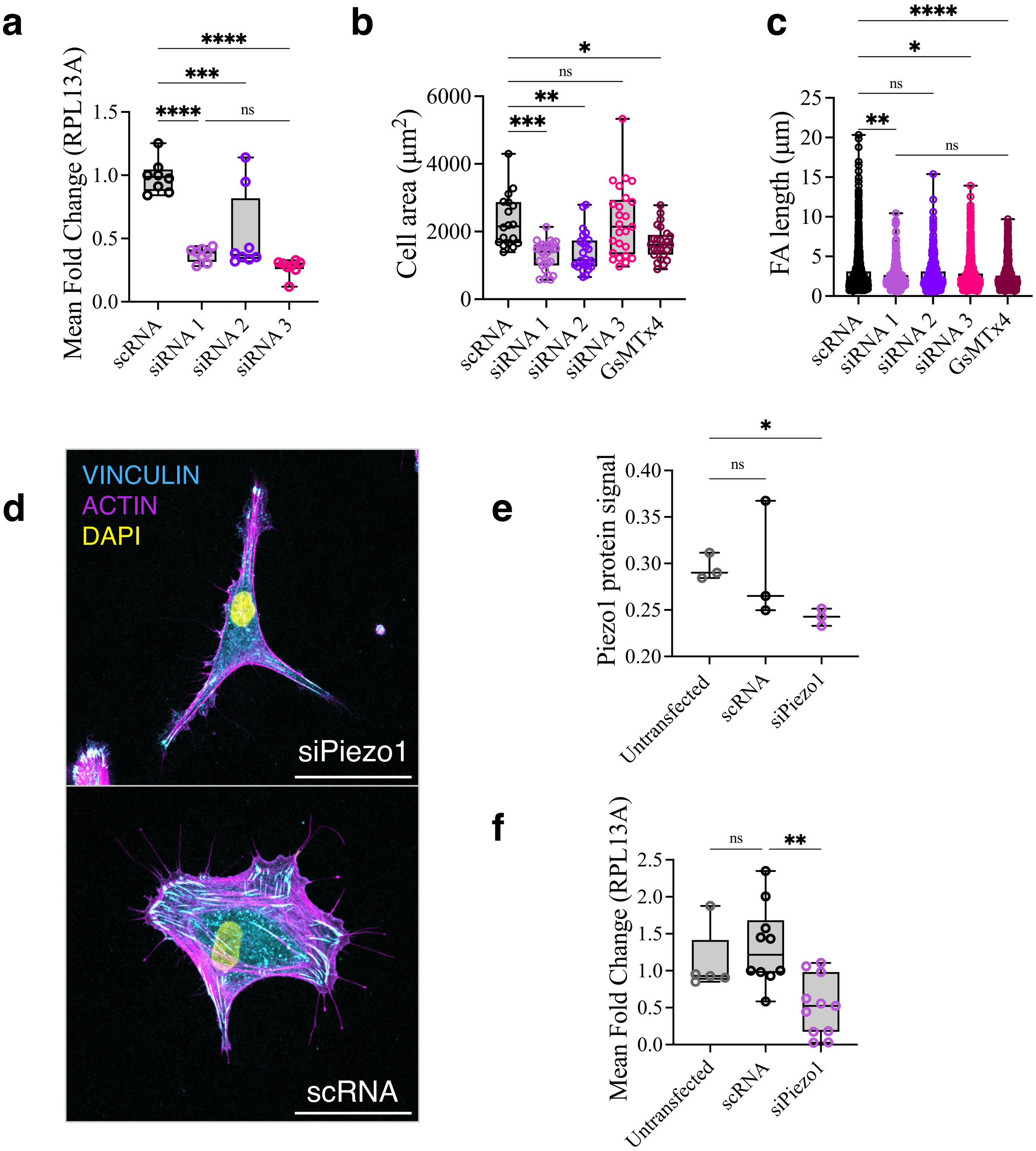

Supplementary Data Fig. 2.

(**a**) RT-qPCR of Piezo1 siRNA screen on Y201 MSCs. Data shown as mean fold change normalized to internal control RPL13A and to scRNA control, plotted as min to max box plot, showing all experimental data points. n = 8 technical replicates from N = 4 independent experiments. P values: **** < 0.0001, *** = 0.001 and ns = 0.6813. (**b**) Mean cell area from Piezo1 siRNA screen and GsMTx4 (10 μM) 48h culture post transfection. Data shown as min to max box & whiskers plot showing all individual points. From left to right n = 18, 27, 24, 25 and

25 cells from N = 2 individual experiments. P values: * = 0.0443, ns > 0.9999, ** = 0.0016 and *** = 0.0002. (**c**) FA length from Piezo1 siRNA screen and GsMTx4 (10 µM) 48h culture post transfection. Data shown as min to max box & whiskers plot showing all individual points. From left to right n = 1002, 460, 494, 919 and 605 adhesions from a minimum of 18 cells, from N = 2 individual experiments. P values, from top to bottom: **** < 0.0001, * = 0.0115, ns = 0.0986, ** = 0.0025 and ns = 0.8421. (**d**) Representative images of siRNA1 (siPiezo1) and scRNA treated Y201 MSCs cultured on FN coated coverslips. Scale bar 50 µm (**e**) Piezo1 protein signal as quantified from in cell western, shown as individual experiment mean ± SD, N = 3, n ≥ 10,000 cells. P values: ns = 0.5510 and * = 0.0369. (**f**) Mean fold change of Piezo1 gene expression normalised to RPL13A, shown as min to max box & whiskers, from left to right n = 11, 10 and 5 technical replicates from N = 4 experiments and p values: ** = 0.0016 and ns = 0.6482. Statistical analyses were performed using a one-way ANOVA (a, b, c, f) and a Kruskal-Wallis test in (e).

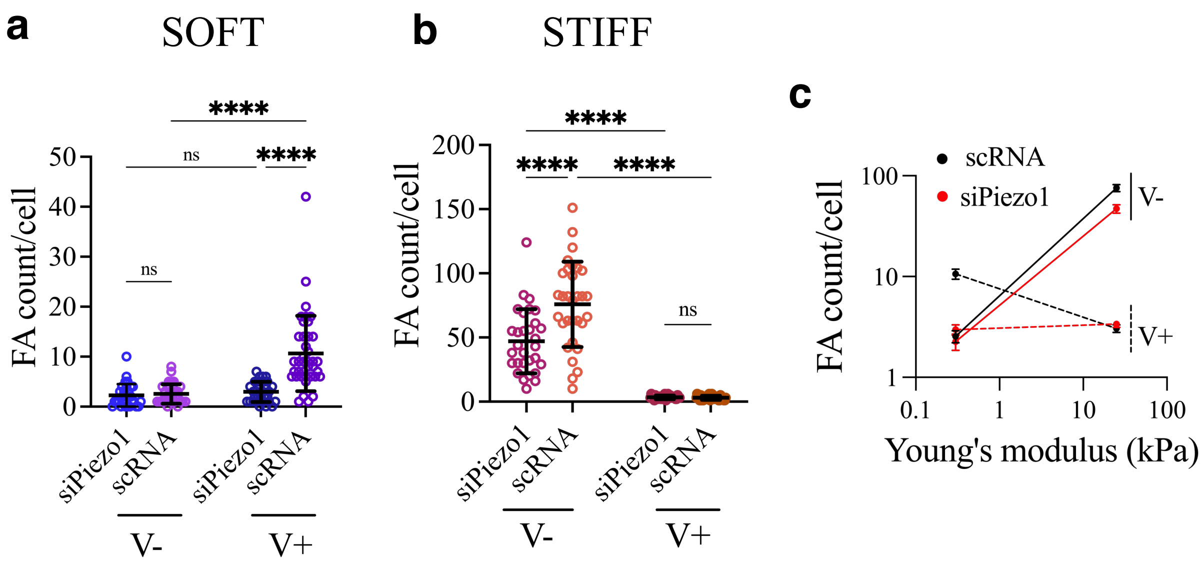

Supplementary Data Fig. 3.

Quantified total focal adhesion per cell (FA count/cell) of the soft (**a**) and stiff (**b**) hydrogel groups. Dots represent individual cell measurements, data shown as individual points, mean ± SD. In (a) from left to right, n = 30, 31, 32 and 39 cells and p values: ns = 0.9936, ns = 0.9202 and **** < 0.0001; in (b) from left to right n = 30, 31, 31 and 35 cells and p values: **** < 0.0001. Data from N = 3 individual experiments. (**c**) Summary of average FA count per cell ± SEM plotted as a function of stiffness for scRNA (black), siPiezo1 (red), V- (continuous line) and V+ (dashed line) conditions. Statistical analyses were performed using a two-way ANOVA.

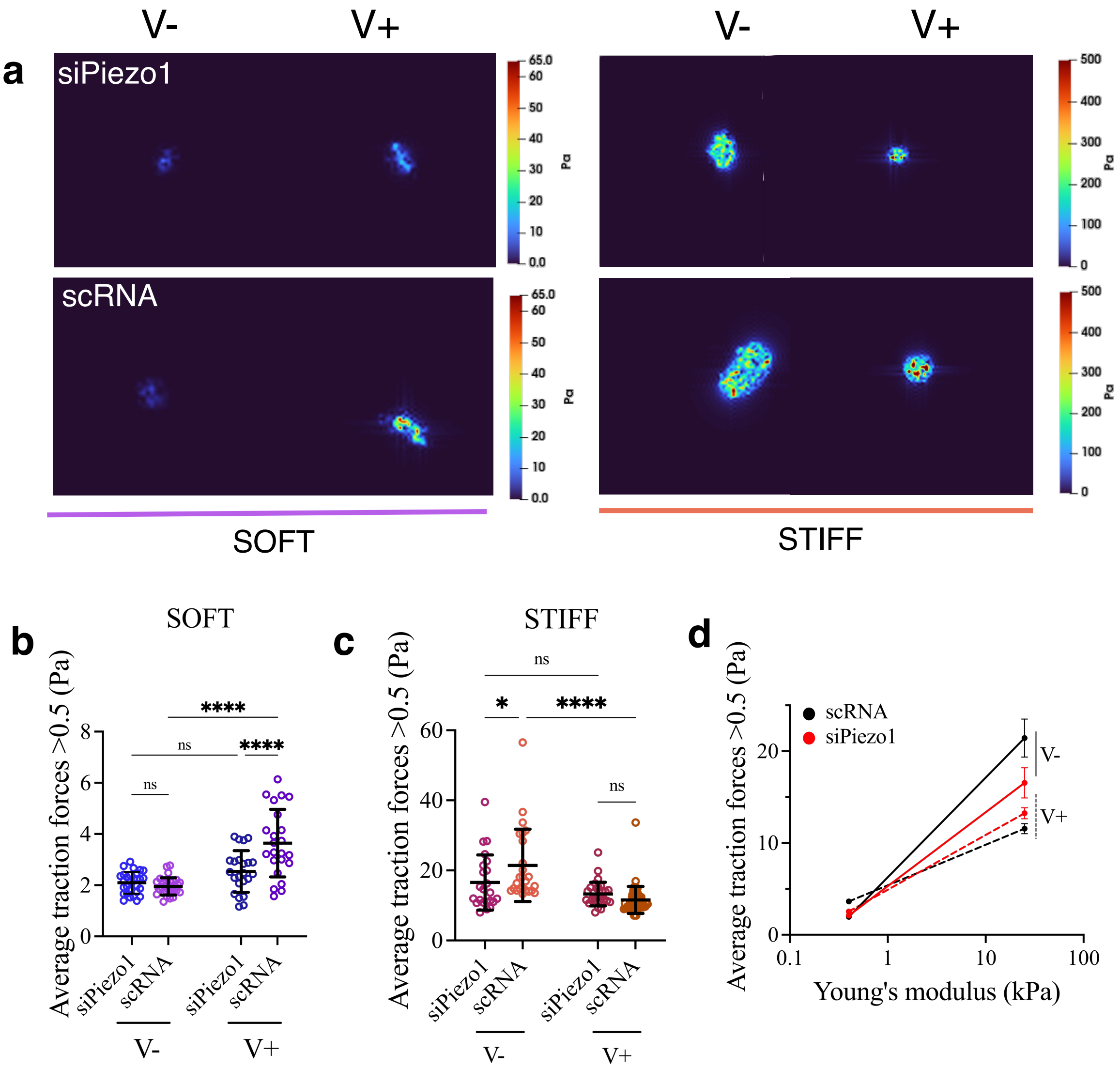

Supplementary Data Fig. 4.

Representative colour maps of traction forces applied by siPiezo1 (top) and scRNA (bottom) cells on soft (left) and stiff (right) hydrogel groups. Average traction forces measured on siPiezo1 and scRNA Y201 cells cultured on the soft (**b**) and stiff (**c**) hydrogel groups. In (b) from left to right, n = 29, 30, 23 and 23 cells and p values: ns = 0.8917, ns = 0.1834 and **** < 0.0001; In (c) from left to right, n = 23, 25, 31 and 50 cells and p values: * = 0.0418 , ns = 0.2336, **** < 0.0001 and ns = 0.6465. Data from N = 2 independent experiments. (**l**) Summary of average traction forces ± SEM plotted as a function of stiffness for all experimental conditions. Statistical analyses were performed using a two-way ANOVA test.

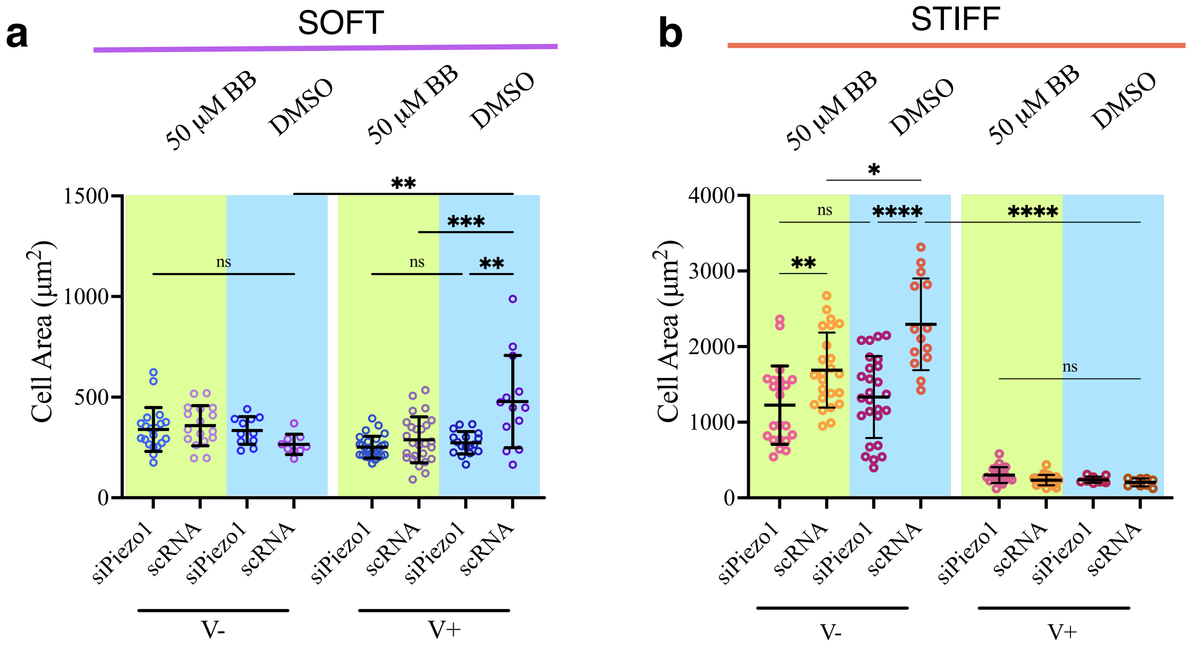

Supplementary Data Fig. 5.

Quantified cell area in cells with Blebbistatin (50 μM BB) treatment or without (DMSO) in the soft (**a**) and (**b**) stiff group. In (a) from left to right, n = 20, 17, 11, 9, 27, 26, 17, 13 cells and p values: ns = 0.7030, ** = 0.0013, ns > 0.9999, *** = 0.0004 and ** = 0.0061; In (b) from left to right, n = 22, 24, 26, 14, 22, 24, 11 and 9 cells and p values: ** = 0.0012, ns = 0.9994, * = 0.0143, **** < 0.0001 and ns = 0.9609. Data from N = 2 independent experiments. Data shown as individual points, mean ± SD. Blue and green shaded bars represent blebbistatin and DMSO treatment conditions, respectively. Statistical analyses were performed using a two-way ANOVA test.

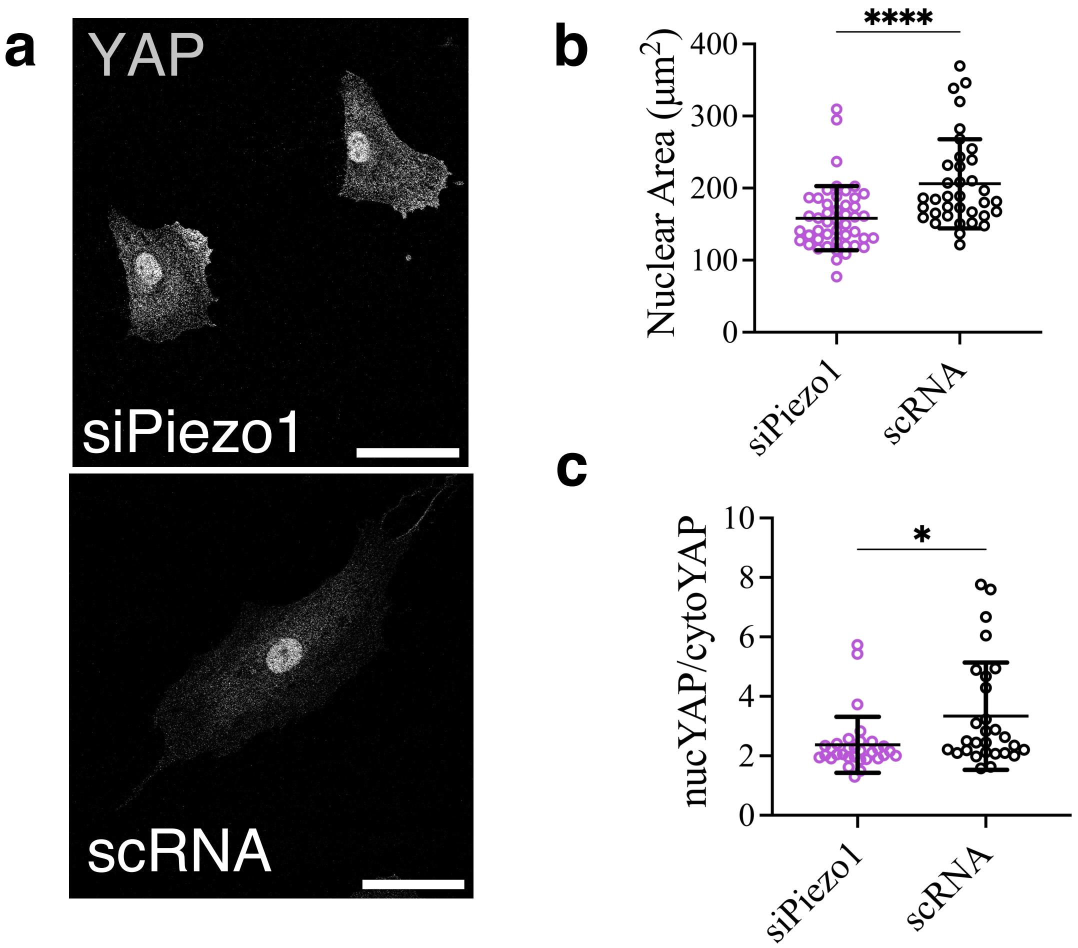

Supplementary Data Fig. 6.

(**a**) Representative images of YAP in (top to bottom) siPiezo1 and scRNA Y201 MSCs cultured on fibronectin coated glass coverslips for 48h. (**b**) Quantified nuclear area shown as μm^2. From left to right n = 47 and 36 cells from N = 3 independent experiments. P value: **** < 0.0001(**c**) Quantified nuclear/cytoplasmic YAP ratio. From left to right, n = 32 and 28 cells from N = 3 independent experiments. P value: * = 0.0102. Data shown as individual values, mean ± SD. Statistical analyses were performed using an unpaired t-test.

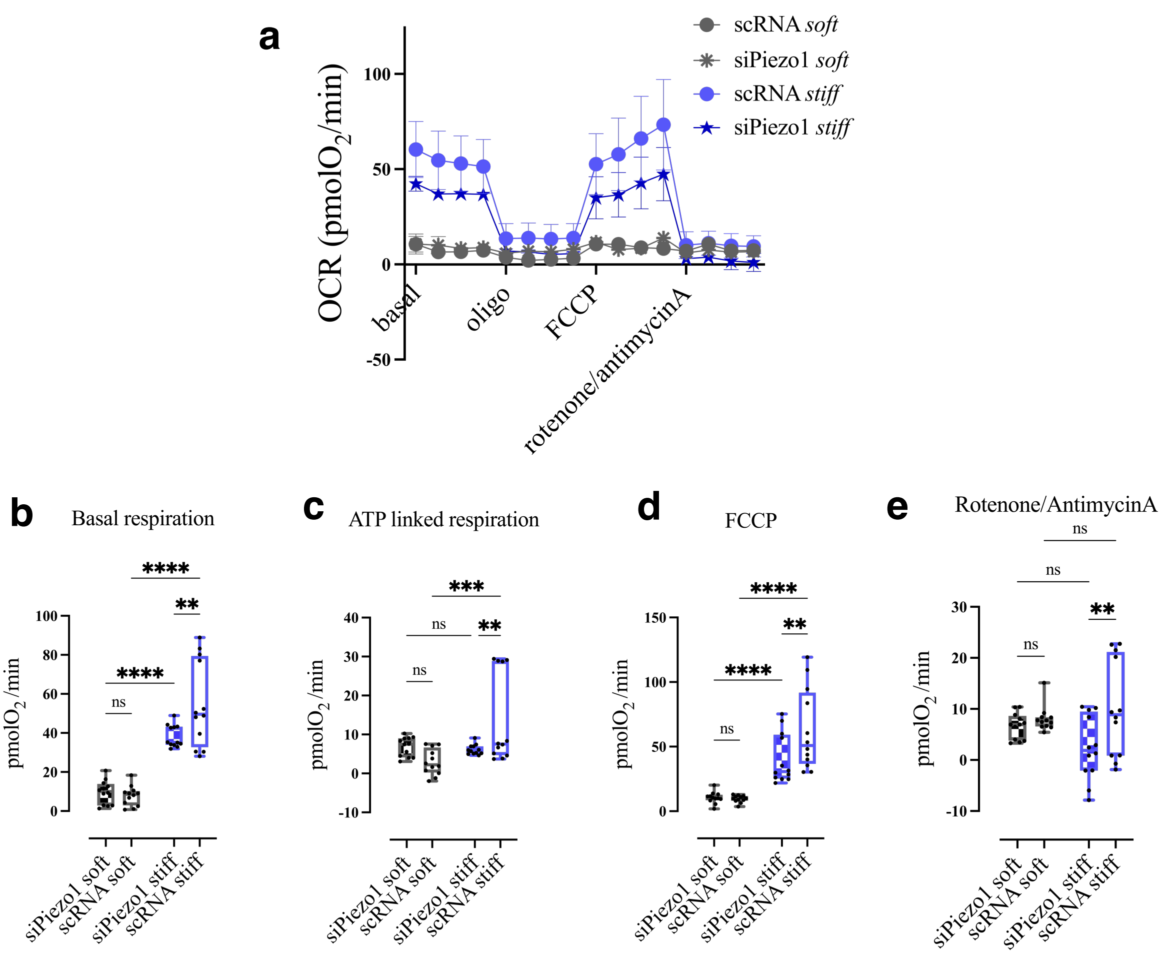

Supplementary Data Fig. 7.

(**a**) Oxygen Consumption Rate (OCR) profile determined with an extracellular flux analyser of siPiezo1 and scRNA cells cultured as cell monolayers on soft (E ~ 200 Pa) and stiff (E ~ 1GPa) Matrigel coated wells. Oligomycin (oligo, 0.8 µM), FCCP (0.9 µM), rotenone (1 µM) plus antimycin A (1 µM) were used to determine the basal respiration, ATP-coupled respiration, maximal respiratory capacity, and non-mitochondrial oxygen consumption, respectively. Data shown as mean ± SEM and represents two independent experiments (N=2) each with n = 4 technical repeats. (**b**) Average respiration rates in the basal respiration phase of the OCR measurements. P values, from top to bottom: **** < 0.0001, ** = 0.0030, **** <0.0001 and ns = 0.7360. (**c**) Average respiration rate for the ATP linked respiration phase (after oligomycin addition). P values, from top to bottom: *** = 0.0003, ** = 0.0075, ns = 0.7827 and ns = 0.1439. (**d**) Average maximal respiration capacity respiration rates after FCCP treatment. P values, from top to bottom: **** < 0.0001, ** = 0.0021, **** < 0.0001 and ns = 0.8951. (**e**) Average non-mitochondrial respiration rates after Rotenone/Antimycin A treatment. P values, from top to bottom: ns = 0.4405, ns = 0.1124, ** = 0.0059 and ns = 0.5994. Data shown as Min. to Max. Box and Whiskers plot with all points shown. A two-way ANOVA statistical test followed by a Fisher's Least Significant Difference (LSD) test was performed.

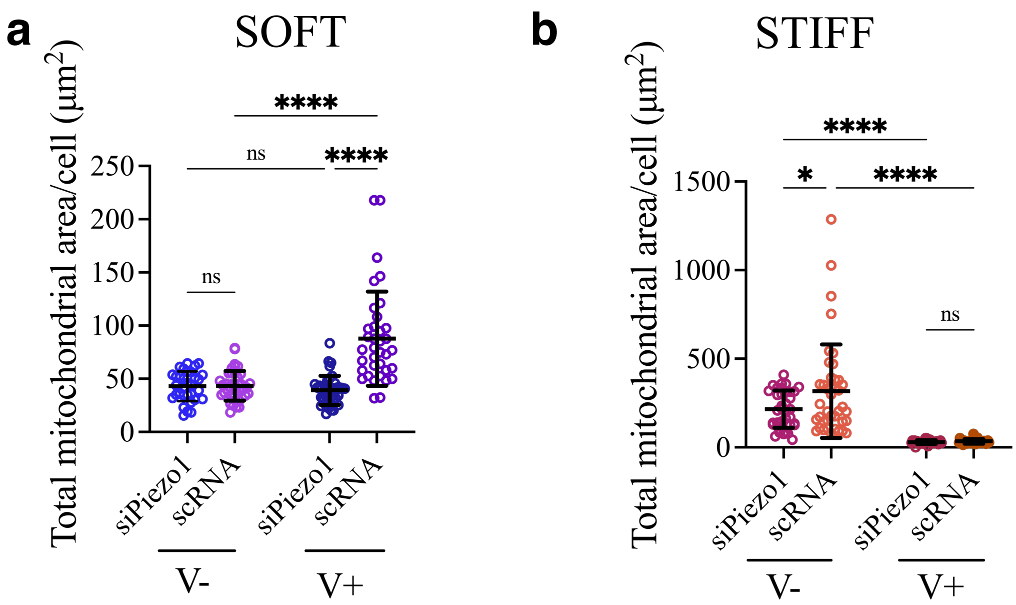

Supplementary Data Fig. 8.

Quantified total mitochondrial area per cell of the soft (**a**) and stiff (**b**) hydrogel groups. In (a) from left to right, n = 32, 40, 40, 36 and p values: ns = 0.9773, ns = 0.6865 and **** < 0.0001; in (b) from left to right, n = 38, 41, 33, 32 and p values: * = 0.0209, **** < 0.0001 and ns = 0.9962. Data from N = independent experiments. Data shown as individual points, mean ± SD. Statistical analyses were performed using a two-way ANOVA test.

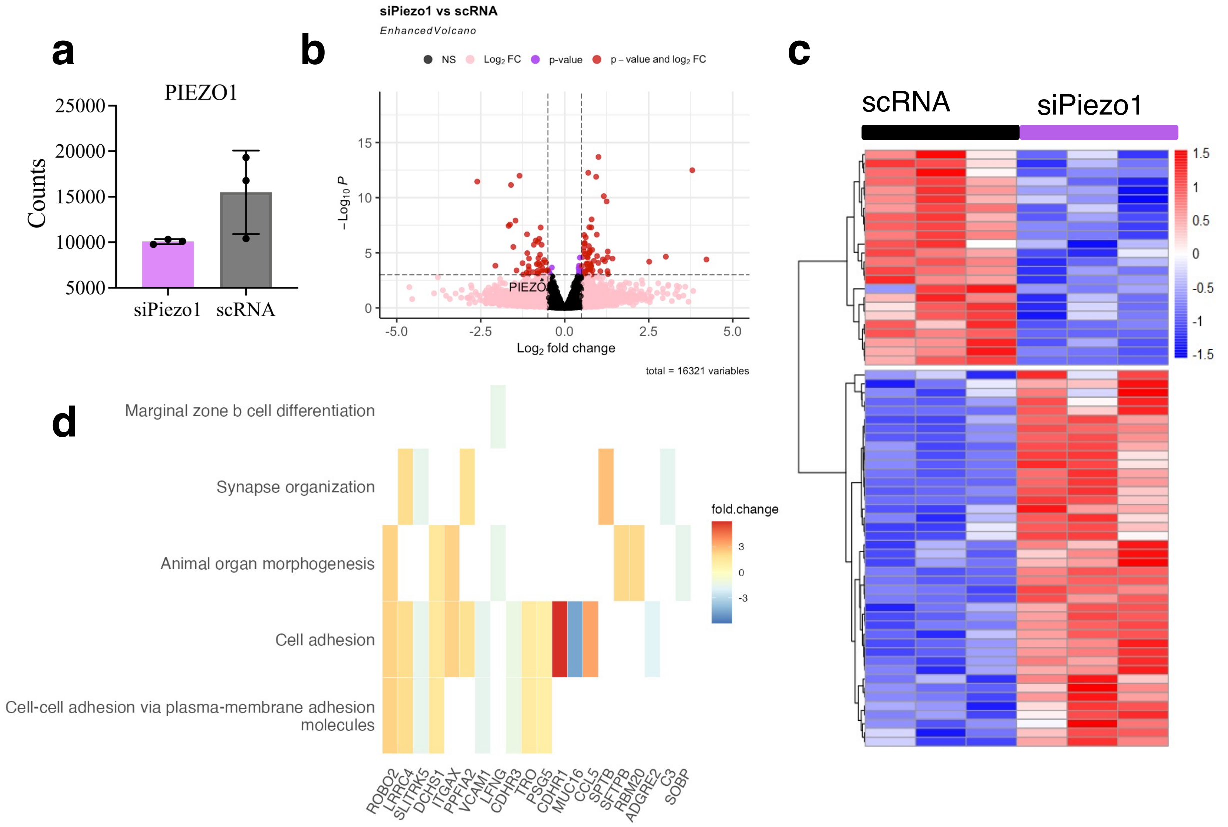

Supplementary Data Fig. 9. (**a**) Normalised Piezo1 gene counts RNAseq data from siPiezo1 and scRNA cells. From N = 3 independent samples. (**b**) Volcano plot of statistical significance (-Log10P) vs fold change (Log_2 fold change) for siPiezo1 vs scRNA comparison. Each dot represents each individual gene. Piezo1 is highlighted on the table, with a fold change of ~-0.6. A total of 16321 genes were used to create the plot. (**c**) Heatmap of scRNA and siPiezo1 MSC differentially expressed genes ($p < 0.05$) from DESeq2 analysis. (**d**) Heatmap of enriched results from Over-representation analysis (ORA) that encompass the most DE genes in the siPiezo1 vs scRNA comparison.

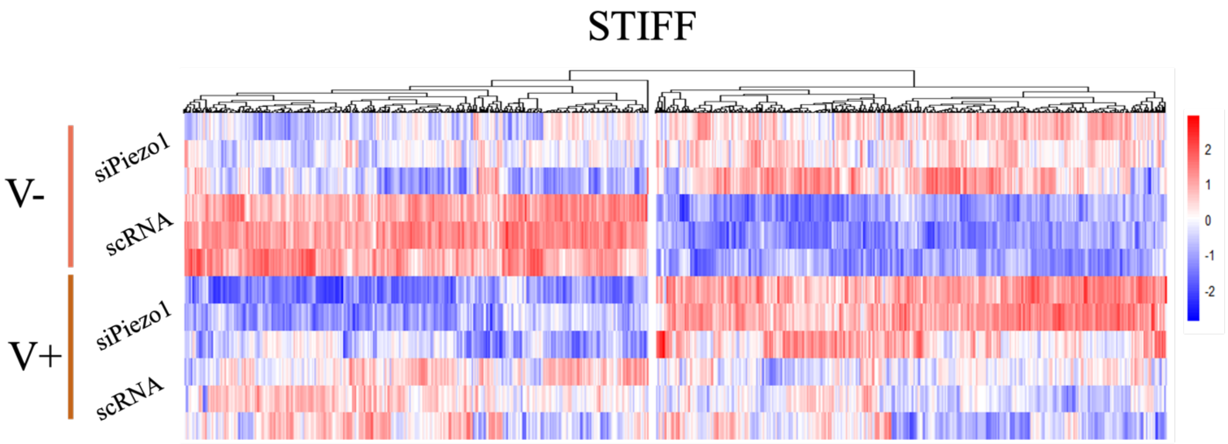

Supplementary Fig. 10. Heatmap for Stiff group genes from DESeq2 analysis. A total of 731 differentially expressed genes are shown, $p < 0.05$. Each row represents an independent sample.

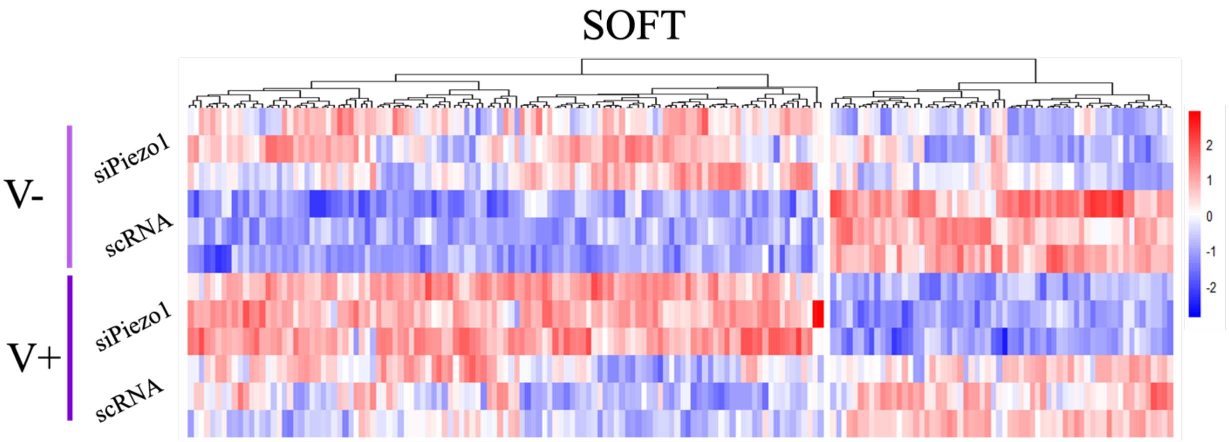

Supplementary Fig. 11. Heatmap for Soft group genes from DESeq2 analysis. A total of 177 differentially expressed genes are shown, $p < 0.05$. Each row represents an independent sample.

Supplementary note 1

Computational model implementation and parameters

To implement the computational clutch model for a viscoelastic substrate, we used our previously described model as a reference [1,] and incorporated a standard linear solid (SLS) model for the substrate instead of a spring (**Fig. 2, e**). The SLS model consists of two springs of stiffness k_1 and k_2, and a dashpot with viscosity η, arranged in series with k_2. The response of an SLS substrate is characterized by the instantaneous stiffness $k_0 = k_1 + k_2$, longterm stiffness $k_\infty = k_1$, and the relaxation time constant $\tau = \eta/k_2$. With that, the constitutive equation for the substrate is given by:

$$F_{\mathrm{sub}}(t) + \tau \frac{dF_{\mathrm{sub}}(t)}{dt} = k_\infty x_{\mathrm{sub}}(t) + k_0 \tau \frac{dx_{\mathrm{sub}}(t)}{dt} \qquad (1)$$

where F_{sub} and x_{sub} are the substrate force and position, respectively.

The Monte Carlo simulation considers a given number of ligands on the substrate n_l, to which integrins will bind. When bound, all ligands are connected to the substrate in parallel. The simulation starts with all ligands unbound, in which case the myosin motors are unloaded and move the actin bundle with a constant rearward speed v_u towards the cell center. At each time step (5 ms), unbound ligands can bind to the substrate with an association rate k_{on} while bound ligands can dissociate with a rate of k_{off}. $k_{\mathrm{on}} = k_{\mathrm{ont}} d_{\mathrm{int}}$, where k_{ont} is the true binding rate characterizing each integrin-ligand bond, and d_{int} is the density of integrins on the cell membrane. After every time step, the substrate position can be determined by applying the force balance between substrate and ligands (clutches) and discretizing the derivates as:

$$x_{\mathrm{sub}}(t) = \frac{\left(1 + \frac{\tau}{\Delta t}\right) k_{\mathrm{clutch}} \sum_{\mathrm{bound}} x_{\mathrm{clutch},i}(t) - \frac{\tau}{\Delta t} F_{\mathrm{sub}}(t - \Delta t) + \frac{k_0 \tau}{\Delta t} x_{\mathrm{sub}}(t - \Delta t)}{k_\infty + \frac{k_0 \tau}{\Delta t} + \left(1 + \frac{\tau}{\Delta t}\right) k_{\mathrm{clutch}} N_{\mathrm{bound}}} \qquad (2)$$

Where $x_{\mathrm{clutch},i}(t)$ is the position of each ligand (clutch) and k_{clutch} is clutch stiffness. The total force F_{sub} is calculated as

$$F_{\mathrm{sub}}(t) = \sum F_{\mathrm{clutch},\,i}(t) \qquad (3)$$

where the force on each ligand is given by:

$$F_{\text{clutch}, i}(t) = k_{\text{clutch}}\left(x_{\text{clutch}, i}(t) - x_{\text{sub}}(t)\right) \quad (4)$$

The ligand position is continuously updated by the myosin motors, which pull on the ligands at a seed v, determined by:

$$v = v_{\text{u}}(1 - \frac{F_{\text{sub}}}{n_{\text{m}} F_{\text{m}}}) \quad (5)$$

Where n_{m} is the total number of myosin motors, and F_{m} is the stall force of a single motor. Reinforcement was modelled by permitting the clutch to unfold with rate k_{uf}, assumed to correspond to talin unfolding [2]. Once the clutch unfolds, vinculin is allowed to bind at a rate $k_{\text{v,on}}$, unless refolding occurs earlier with a rate of k_{f}. Upon successful vinculin binding, d_{add} integrins are added to the system. To maintain a stable integrin population over time, integrins also need to be removed, which happens when the clutch unbinds. The amount of integrins is limited by a lower bound d_{int}, corresponding to the start condition.

To capture the influence of the Piezo1, we increased k_{off} by a factor p_{knock} to simulate a Piezo knockdown, thereby reducing the integrin binding affinity [3]. Integrin removal was increased by this factor as well.

To model viscoelasticity, we used the experimentally obtained values of τ. For k_0 and k_∞, we converted experimental values (in units of Pa) to model parameters (in units of N/m) by assuming a given radius of adhesion a, as done previously [1]. Further, we also considered that in experiments, k_0 between the elastic and viscoelastic case were designed to be equal as measured during nanoindentation experiments **(Fig. 1, b).** However, the contribution of viscosity to the material's response depends on the strain rate (or equivalently, the loading rate). In experiments, the area on which force is applied is determined by the nanoindenter probe diameter (27.5 μm), whereas the area of integrin adhesions applying force is likely to be smaller. Thus, we can assume that strain rates applied by cells are significantly higher compared to the nanoindenter case. At higher strain rates, the dashpot element in the viscoelastic model resists deformation more strongly, contributing to an increased instantaneous stiffness k_0. We thus assumed an increase by a factor of 10 and maintained the observed experimental value for k_∞.

Supplementary tables

Table S1.

Model parameters.

All parameter values, except the cell type specific number of ligands, are of the same order as those employed in previous simulations that considered elasticity rather than viscoelasticity [1,2,4].

Parameter	meaning	Value	Origin
n_m	Number of myosin motors	300	Adjusted
n_l	Number of ligands	3000	Adjusted
F_m	Myosin motor stall force	2 pN	[5]
v_u	Unloaded myosin motor velocity	110 nm/s	[1]
d_{int}	Initial integrin density on the membrane	100/μm^2	Adjusted
k_{ont}	True binding rate	2.1x10^{-5} um^2/s	Adjusted, of the order of values reported for αIIBβ3 [6]
k_{off}	Unbinding rate, scaling factor applied to force curve reported in [7]	0.15	Adjusted, catch bond dependency from [7]
k_{uf}	Talin unfolding rate	Slip bond	[8]
k_{f}	Talin refolding rate	Slip bond	[8]
FR	Fraction of force experienced by talin	0.073	[2]
k_{c}	Clutch stiffness	1nN/nm	[9]
$k_{\mathrm{v,on}}$	Vinculin binding rate	1x10^4 s^{-1}	Adjusted
τ	Relaxation time of substrates	2s	As measured
k_{∞}/k_0	Determines magnitude of substrate relaxation	0.08	Adjusted, based on measurement
p_{knock}	Fold increase in k_{off} after Piezo knockdown	1.15	Adjusted
d_{add}	Integrins added after each reinforcement event	15/μm^2	Adjusted
a	Radius of adhesion	600 nm	Adjusted

Table S2.
Hydrogel formulations.

Reagent	Soft V-	Soft V+	Stiff V-	Stiff V+
**Aam (%)**	3	3	15	35
**BisAam (%)**	0.06	0.06	0.1	0.0124
**40% Aam (µl)**	75	75	375	875
**2% BisAam (µl)**	30	30	50	6.2
**mQ H2O (µl)**	812.5	/	565	108.8
**TEMED 1.5%/100% (µl)**	62.5 (1.5%)	62.5 (1.5%)	2.5 (100%)	2.5 (100%)
**APS 5%/10% (µl)**	20 (5%)	20 (5%)	7.5 (10%)	7.5 (10%)
**Linear Aam (µl)**	/	812.5	/	/
**Total volume (µl)**	**1000**	**1000**	**1000**	**1000**

Table S3.

List of antibodies and other reagents used for immunodetection and qPCR

Reagent	Provider	Dilution
**Alexa Fluor™ 488 Phalloidin**	Thermo (A12379)	1:250
**Anti-YAP Antibody (63.7)**	Santa Cruz (sc-101199)	1:100
**Anti-Fibronectin antibody**	Sigma (F3648)	1:200
**Anti-Vinculin antibody**	Sigma (V9264)	1:400
**Anti-TOMM20**	Abcam (ab186735)	1:200
**Anti-Piezo1**	Sigma (AMAB91589)	1:100
**Cy™3 AffiniPure Rabbit Anti-Mouse IgG (H+L)**	Jackson ImmunoResearch (315-165-003)	1:200
**Donkey anti-Rabbit IgG (H+L) Alexa Fluor™ 488**	Invitrogen (A-21206)	1:250
**CellTag™ 700 Stain for In-Cell Western™ Assays**	Li-Cor (926-41090)	1:500
**IRDye® 800CW Goat anti-Mouse IgG Secondary Antibody**	Li-Cor (926-32210)	1:500
**Piezo1 F' Primer**	Sigma	100 µM stock 5'-TGAAGCGGGAGCTCTACAAC-3'
**Piezo1 R' Primer**	Sigma	100 µM stock 5'-TCTCGTTGGGATACTCCACA-3'
**RPL3 F' Primer**	Eurofins	100 µM stock 5'-CTCAAGGTGTTTGACGGCATCC-3'
**RPL3 R' Primer**	Eurofins	100 µM stock 5'-TACTTCCAGCCAACCTCGTGAG-3'

Table S4.

Representative genes for each functional group collected from the MSigC2 database.

Integrin	Actin	YAP	Mitochondria	Contractility
ITGA2	CDC42	YAP1	VDAC1	PPP1R12A
ITGA1	PFN1	TEAD1	SDHB	MYH11 (ENSG0000013392)
ITGA3	ARPC1B	TEAD4	TOMM40	ROCK2
VCL	WASF2	TEAD2	COX411	TAGLN
TLN1	FLNA	LATS1	NDUFA9	ROCK1
ITGB1	ACTB	NF2	ATP5MC1	ACTA2
ITGA5	MYH9	WWTR1	UQCRC1	CNN1
ITGB3	RAC1	LATS2	NDUFS1	TPM1
ITGA6	CFL1	MOB1A	ATP5F1A	MYL9
PTK2	ACTG1		NDUFS1	MYH11 (ENSG00000276480)

Supplementary references

1. Elosegui-Artola, A. *et al.* Rigidity sensing and adaptation through regulation of integrin types. *Nat Mater* **13**, 631–637 (2014).
2. Elosegui-Artola, A. *et al.* Mechanical regulation of a molecular clutch defines force transmission and transduction in response to matrix rigidity. *Nat Cell Biol* **18**, 540–548 (2016).
3. Cheng, D., Wang, J., Yao, M. & Cox, C. D. Joining forces: crosstalk between mechanosensitive PIEZO1 ion channels and integrin-mediated focal adhesions. *Biochem Soc Trans* (2023) doi:10.1042/BST20230042.
4. Oria, R. *et al.* Force loading explains spatial sensing of ligands by cells. *Nature* **552**, 219–224 (2017).
5. Molloy, J. E., Burns, J. E., Kendrick-Jones, J., Tregear, R. T. & White, D. C. S. Movement and force produced by a single myosin head. *Nature* **378**, 209–212 (1995).
6. Litvinov, R. I. *et al.* Resolving Two-dimensional Kinetics of the Integrin αIIbβ3-Fibrinogen Interactions Using Binding-Unbinding Correlation Spectroscopy. *Journal of Biological Chemistry* **287**, 35275–35285 (2012).
7. Kong, F., García, A. J., Mould, A. P., Humphries, M. J. & Zhu, C. Demonstration of catch bonds between an integrin and its ligand. *Journal of Cell Biology* **185**, 1275–1284 (2009).
8. Yao, M. *et al.* Mechanical activation of vinculin binding to talin locks talin in an unfolded conformation. *Sci Rep* **4**, 4610 (2014).
9. Riveline, D. *et al.* Focal Contacts as Mechanosensors. *J Cell Biol* **153**, 1175–1186 (2001).
