# Supplementary material for: Piezo1 regulates the mechanotransduction of soft matrix viscoelasticity
Source: Nat Commun. 2025 Oct 15;16:9155. doi: 10.1038/s41467-025-64185-5 (PMC12528374; doi:10.1038/s41467-025-64185-5)
Supplement: Supplementary file 2 — Reporting summary [file 41467_2025_64185_MOESM2_ESM.pdf]

nature portfolio

Corresponding author(s): Manuel Salmeron-Sanchez

Last updated by author(s): Jul 31, 2025

Reporting Summary

Nature Portfolio wishes to improve the reproducibility of the work that we publish. This form provides structure for consistency and transparency in reporting. For further information on Nature Portfolio policies, see our Editorial Policies and the Editorial Policy Checklist.

Statistics

For all statistical analyses, confirm that the following items are present in the figure legend, table legend, main text, or Methods section.

n/a	Confirmed	
☐	☒	The exact sample size (*n*) for each experimental group/condition, given as a discrete number and unit of measurement
☐	☒	A statement on whether measurements were taken from distinct samples or whether the same sample was measured repeatedly
☐	☒	The statistical test(s) used AND whether they are one- or two-sided<br>*Only common tests should be described solely by name; describe more complex techniques in the Methods section.*
☐	☒	A description of all covariates tested
☐	☒	A description of any assumptions or corrections, such as tests of normality and adjustment for multiple comparisons
☐	☒	A full description of the statistical parameters including central tendency (e.g. means) or other basic estimates (e.g. regression coefficient) AND variation (e.g. standard deviation) or associated estimates of uncertainty (e.g. confidence intervals)
☐	☒	For null hypothesis testing, the test statistic (e.g. *F*, *t*, *r*) with confidence intervals, effect sizes, degrees of freedom and *P* value noted<br>*Give P values as exact values whenever suitable.*
☒	☐	For Bayesian analysis, information on the choice of priors and Markov chain Monte Carlo settings
☒	☐	For hierarchical and complex designs, identification of the appropriate level for tests and full reporting of outcomes
☒	☐	Estimates of effect sizes (e.g. Cohen's *d*, Pearson's *r*), indicating how they were calculated

Our web collection on statistics for biologists contains articles on many of the points above.

Software and code

Policy information about availability of computer code

Data collection	As detailed in the methods section, microscopy data was collected primarily with micromanager and ZEN blue. Material characterisation was performed primarily with the Chiaro system (Optics11) and RheoPlus (AntonParr). In cell western data was obtained from Odyssey system. OCR measurements were extracted from a Seahorse Bioscience Flux analyser. RNAseq library was prepared with a NextSeq2000 system.
Data analysis	As described in the methods section, data was analysed primarily using FIJI. Nanotopography analysis and plots were performed in the beta version of the Prova software (Optics11). Plotting and statistical analysis was performed using Prism Graph Pad 9 and R studio.

For manuscripts utilizing custom algorithms or software that are central to the research but not yet described in published literature, software must be made available to editors and reviewers. We strongly encourage code deposition in a community repository (e.g. GitHub). See the Nature Portfolio guidelines for submitting code & software for further information.

Data

Policy information about availability of data

All manuscripts must include a data availability statement. This statement should provide the following information, where applicable:

- Accession codes, unique identifiers, or web links for publicly available datasets
- A description of any restrictions on data availability
- For clinical datasets or third party data, please ensure that the statement adheres to our policy

All data are available in the main text or supplementary materials. Analysis scripts are available upon request. The transcriptomics datasets from this work have been deposited to Gene Expression Omnibus with the accession codes GSE288423, GSE288464 and GSE288465.

Research involving human participants, their data, or biological material

Policy information about studies with human participants or human data. See also policy information about sex, gender (identity/presentation), and sexual orientation and race, ethnicity and racism.

Reporting on sex and gender	Cells used in this study are Y201 MSCs developed at the University of York ((https://pubmed.ncbi.nlm.nih.gov/26070611/)
Reporting on race, ethnicity, or other socially relevant groupings	NA
Population characteristics	NA
Recruitment	NA
Ethics oversight	NA

Note that full information on the approval of the study protocol must also be provided in the manuscript.

Field-specific reporting

Please select the one below that is the best fit for your research. If you are not sure, read the appropriate sections before making your selection.

☒ Life sciences ☐ Behavioural & social sciences ☐ Ecological, evolutionary & environmental sciences

For a reference copy of the document with all sections, see nature.com/documents/nr-reporting-summary-flat.pdf

Life sciences study design

All studies must disclose on these points even when the disclosure is negative.

Sample size	No statistical method was used to determine sample size. Sample size was chosen based on previous experience and standards in the field. Each data point represents an individual cell, except where stated otherwise in the figure caption. All n are listed in the text in figure captions.
Data exclusions	No data were excluded from analysis
Replication	Experiments were replicated independently three times, unless otherwise stated in the figure legends (N).
Randomization	Due to the nature of this research, randomization is not appropriate as assignment to categories must be exact therefore no systematic randomization was required.
Blinding	Investigators were not blinded during experiment or analysis, as blinding was not possible since experimental conditions were evident from image data being analyzed.

Reporting for specific materials, systems and methods

We require information from authors about some types of materials, experimental systems and methods used in many studies. Here, indicate whether each material, system or method listed is relevant to your study. If you are not sure if a list item applies to your research, read the appropriate section before selecting a response.

Materials & experimental systems

n/a	Involved in the study
☐	☒ Antibodies
☐	☒ Eukaryotic cell lines
☒	☐ Palaeontology and archaeology
☒	☐ Animals and other organisms
☒	☐ Clinical data
☒	☐ Dual use research of concern
☒	☐ Plants

Methods

n/a	Involved in the study
☒	☐ ChIP-seq
☒	☐ Flow cytometry
☒	☐ MRI-based neuroimaging

Antibodies

Antibodies used

Anti-YAP Antibody (63.7) (Santa Cruz (sc-101199))
Anti-Fibronectin antibody (Sigma (F3648))
Anti-Vinculin antibody (Sigma (V9264))
Anti-TOMM20 (Abcam (ab186735))
Anti-Piezo1 (Sigma (AMAB91589))

Validation

All commercial antibodies used in this study have been validated by the manufacturers from whom they were purchased.

Eukaryotic cell lines

Policy information about cell lines and Sex and Gender in Research

Cell line source(s)

Y201 MSCs were sourced from Paul Genever's group in the University of York

Authentication

No euthentication was performed

Mycoplasma contamination

All cells were negative for microplasma contamination (tested with N-Garde kit , Euroclone EMK090020)

Commonly misidentified lines
(See ICLAC register)

NA

Plants

Seed stocks

NA

Novel plant genotypes

NA

Authentication

NA
